# Mutations of Glucose-6-Phosphate Dehydrogenase Durham, Santa-Maria and A+ Variants Are Associated with Loss Functional and Structural Stability of the Protein

**DOI:** 10.3390/ijms161226124

**Published:** 2015-12-02

**Authors:** Saúl Gómez-Manzo, Jaime Marcial-Quino, America Vanoye-Carlo, Sergio Enríquez-Flores, Ignacio De la Mora-De la Mora, Abigail González-Valdez, Itzhel García-Torres, Víctor Martínez-Rosas, Edgar Sierra-Palacios, Fernando Lazcano-Pérez, Eduardo Rodríguez-Bustamante, Roberto Arreguin-Espinosa

**Affiliations:** 1 Laboratorio de Bioquímica Genética, Instituto Nacional de Pediatría, Mexico D.F. 04530, Mexico; sergioenr@gmail.com (S.E.-F.); ignaciodelamora@yahoo.com.mx (I.D.M.-D.M.); itzheltorres@hotmail.com (I.G.-T.); razer_45r@hotmail.com (V.M.-R.); 2 CONACYT Research Fellow—Instituto Nacional de Pediatría, Mexico D.F. 04530, Mexico; 3 Laboratorio de Neurociencias, Instituto Nacional de Pediatría, Mexico D.F. 04530, Mexico; america_vc@yahoo.com.mx; 4 Departamento de Biología Molecular y Biotecnología, Instituto de Investigaciones Biomédicas, Universidad Nacional Autónoma de Mexico, Mexico D.F. 04510, Mexico; abigaila@correo.biomedicas.unam.mx; 5 Colegio de Ciencias y Humanidades, Plantel Casa Libertad, UACM, Mexico D.F.09620, Mexico; edgar.sierra@uacm.edu.mx; 6 Departamento de Química de Biomacromoléculas, Instituto de Química, Universidad Nacional Autónoma de Mexico, México, D.F. 04510, Mexico; ferlaz@hotmail.com (F.L.-P.); e-rodriguez-bustamante@ciencias.unam.mx (E.R.-B.); arrespin@unam.mx (R.A.-E.)

**Keywords:** G6PD deficiency, G6PD-variants, recombinant expression, kinetic-properties, stability

## Abstract

Glucose-6-phosphate dehydrogenase (G6PD) deficiency is the most common enzymopathy in the world. More than 160 mutations causing the disease have been identified, but only 10% of these variants have been studied at biochemical and biophysical levels. In this study we report on the functional and structural characterization of three naturally occurring variants corresponding to different classes of disease severity: Class I G6PD Durham, Class II G6PD Santa Maria, and Class III G6PD A+. The results showed that the G6PD Durham (severe deficiency), and the G6PD Santa Maria and A+ (less severe deficiency) (Class I, II and III, respectively) affect the catalytic efficiency of these enzymes, are more sensitive to temperature denaturing, and affect the stability of the overall protein when compared to the wild type WT-G6PD. In the variants, the exposure of more and buried hydrophobic pockets was induced and monitored with 8-Anilinonaphthalene-1-sulfonic acid (ANS) fluorescence, directly affecting the compaction of structure at different levels and probably reducing the stability of the protein. The degree of functional and structural perturbation by each variant correlates with the clinical severity reported in different patients.

## 1. Introduction

The deficiency of glucose-6-phosphate dehydrogenase (G6PD) (EC 1.1.1.49) has been recognized as the most common enzymopathy, because it affects near 400 million people worldwide [1]. The G6PD is an X-linked house-keeping cytosolic enzyme [2] and participates in the first step of the pentose phosphate pathway, catalyzing the conversion of glucose 6-phosphate (G6P) to 6-phosphogluconolactone with the concomitant production of one molecule of nicotinamide adenine dinucleotide phosphate (NADPH). NADPH is involved in redox equilibrium due to its participation in the regeneration of the reduced glutathione (GSH) promoted by the glutathione reductase (GR) enzyme. The G6PD enzyme plays a fundamental role in erythrocytes, due to the maintenance of haemoglobin and other proteins in a reduced state that depends on the glutathione antioxidant system [3].

The G6PD deficiency has a widespread clinical manifestation from asymptomatic to acute or chronic hemolytic states. G6PD deficiency is genetically heterogeneous, with 160 mutations reported to date [4,5]. The G6PD variants are traditionally classified according to their residual enzyme activity and hematologic parameters of patients with G6PD deficiency, ranging from Class I to Class V. Mutations with a Class I phenotype have the most severe clinical manifestation and are usually product of mutations in the exon 10, which codifies for the structural NADP^+^ binding site [6]. In order to understand the molecular basis of the G6PD deficiency, it is important to know how different mutations affect the enzyme’s structure, stability, and function. Nowadays, the effects of only 10% of all recognized variants at the protein level as well as their relationships with the clinical manifestations have been studied.

**Figure 1:**
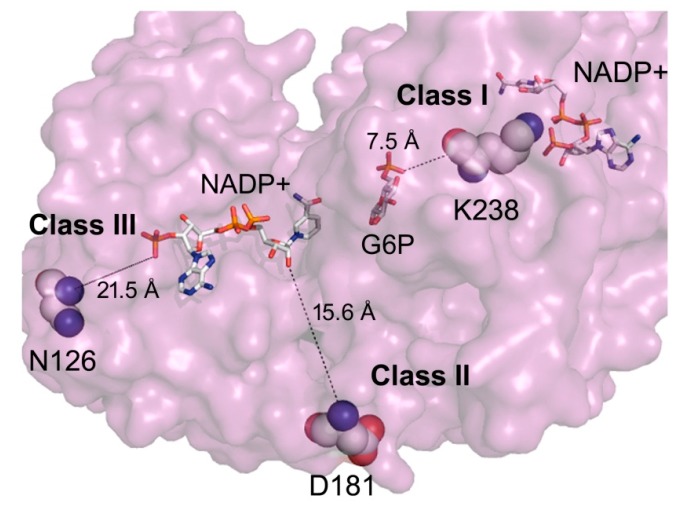
Schematic representation of human G6PD enzyme (PDB code 2BH9) [17]. Schematic representation in cartoon, spheres and sticks of recombinant human G6PD Durham, Santa-Maria and A+ variants in the crystallographic structure of human WT-G6PD derived from PDB code 2BH9. Modeled with the molecular viewer Python Molecular (PyMOL) [18].

Previously, we reported on the structural and functional characterization of three different G6PD variants related to Class I, II and III, finding that the affectation in functional and structural parameters was correlated with a more severe phenotype [7]. In this work, we report the construction, cloning, expression and detailed functional and structural studies of three new clinical variants of G6PD, located in different parts of the protein, and we compare these with the recombinant human WT-G6PD enzyme. The Class I, G6PD Durham involves the substitution of adenine for guanine nucleotide (nt) (A → G) at position 713 (exon 7), resulting in the change of lysine to arginine amino acid residue 238 (K → R) [8,9] (Figure 1), which is near to the structural NADP^+^ site, and is related with severe clinical manifestations as hemolityc anaemia and chronic nonspherocytic hemolityc anaemia. The Class II G6PD Santa Maria variant is related to haemolysis after ingestion of broad beans and involves a mutation in the exon 5 of A → G substitution at nt 376 plus an A → T transversion at the nt 542, causing changes in the 126 (Asn → Asp) and 181 (Asp → Val) amino acid residues [10,11,12] (Figure 1). Finally, the Class III G6PD A+ variant shows an A → G substitution in the exon at nt 376 with a change in the 126 (Asn → Asp) [13,14,15] amino acid residue and is related to an asymptomatic G6PD deficiency form [16]. The locations of each of the mutations mentioned and analyzed in this study are distant from the active site (Figure 1).

## 2. Results

### 2.1. Expression and Purification of Recombinant Human Glucose-6-Phosphate Dehydrogenase (G6PD) Enzymes

**Figure 2:**
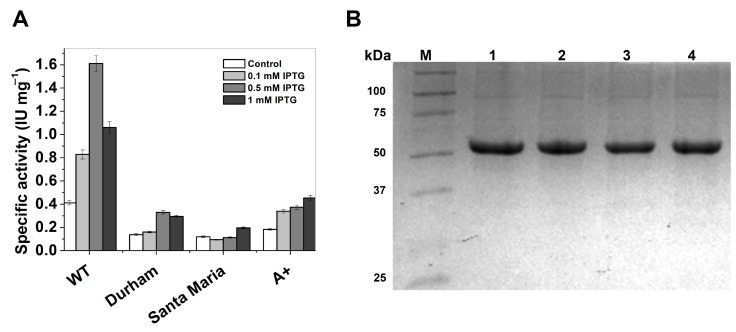
Expression assays and Sodium Dodecyl Sulfate Polyacrylamide Gel Electrophoresis SDS-PAGE analysis of recombinant human G6PDs. (**A**) Specific activity was use as indicative of the expression levels of soluble recombinant protein. The standard deviations represent the values of triplicates samples; (**B**) SDS-PAGE analysis of the purified G6PDs. M: Molecular weight marker from Bio-rad (Broad Range). Line 1: WT. Line 2: Durham. Line 3: Santa-Maria. Line 4: A+. Each lane was loaded with 10 µg of protein, the gel was revealed with Coomassie Brilliant Blue.

The plasmids constructed (pETg-Durham, pETg-Santa-Maria, and pETg-A+) with the desired mutations were used to transform competent *E. coli* BL21(DE3)Δ*zwf*::kan^r^ cells. Optimal expression conditions of soluble proteins WT-G6PD and three variants were determined measuring the specific activity on crude extracts from *E. coli* BL21(DE3)Δ*zwf*::kan^r^ cells. The best overexpression conditions with IPTG and growth temperatures were at 25 °C, because at 37 and 15 °C the specific activities in all cases were lower. For the G6PD Durham enzyme a specific activity of 0.32 IU·mg^−1^ was obtained with 0.5 mM of IPTG and 18 h of incubation; which represents a decrease of 5-fold in the specific activity with respect to WT-G6PD enzyme (Figure 2A). The optimal expression condition for the G6PD Santa-Maria enzyme was obtained with 0.1 mM IPTG and 12 h of incubation, with a specific activity of 0.19 IU·mg^−1^. Finally, the higher expression of soluble G6PD A+ was obtained with 1 mM IPTG and 18 h of incubation; the specific activity was 0.45 IU·mg^−1^. It is noteworthy that even though those mutations are located in different parts of the three-dimensional structure of the WT-G6PD protein, the protein expression was lower than WT-G6PD (1.6 IU·mg^−1^) [12] (Figure 2A).

After purification of recombinant human G6PD enzymes, a single band with 96% of purity was obtained as observed in SDS-PAGE (Figure 2B), which allowed us to do the functional and structural assays for the variants used in this work. We obtained approximately 2 mg of almost pure protein for all the variants.

The yield from the purification of the Class I G6PD Durham variant was approximately 21%, while the Class II G6PD Santa-Maria and Class III G6PD A+ variants gave yields of 31% and 43%, respectively. As expected, the purification yield of the G6PD A+ is very similar to that obtained for the WT-G6PD enzyme. Notably, all the G6PD variants showed a lower yield compared with the WT-G6PD (Table 1).

**Table 1:** Purification summary of the recombinant human Wild-type (WT-G6PD) and the variant enzymes.

G6PD	Total Protein (mg)	Specific Activity (IU·mg^−1^)	Total Activity (IU)	Yield (%)
WT	1.95	224	436	61
Durham	1.15	71	81	21
Santa-Maria	1.41	71	100	32
A+	1.81	114	373	43

The specific activity was measured under standard conditions as described in Materials and Methods.

### 2.2. Functional Characterization of Recombinant Human G6PD Enzymes

#### 2.2.1. Steady-State Kinetics of G6PD Enzymes

Steady-state kinetic parameters were determined for the WT-G6PD and the three clinical G6PD variants. The *K*_mG6P_ values for the G6PD Durham and Santa-Maria variants show more affinity for substrate G6P with respect to WT-G6PD enzyme (24 and 15 µM, respectively) (Table 2). However, the affinity for the catalytic coenzyme NADP^+^ was the same as previously reported for the WT-G6PD (*K*_mG6P_ 6 µM) [7,19,20]. Nonetheless, the *k*_cat_ values obtained for the G6PD Durham (71 s^−1^) and G6PD Santa-Maria (71 s^−1^) (Table 2) showed a loss of catalysis around the 70% for both variants with respect to the WT-G6PD. The *K*_m_ value for the G6PD A+ was twice as high (*K*_mG6P_ = 56, *K*_mNADP+_ = 13 µM, respectively) for both physiological substrates, and the *k*_cat_ value decreased 50% (114 s^−1^) respect to WT-G6PD enzyme (Table 2). Due to the fact that all the variants have lower purification yield and less catalytic efficiency, we performed several trials in order to determine if the mutations have local effects as well as those caused by each point mutation in the tertiary structure.

**Table 2:** Catalytic properties of human G6PD and the variants.

Kinetic Constants	WT-G6PD	Variants		
		Durham	Santa-Maria	A+
*K*_m_ G6P (µM)	38.49	24.77	15.35	56.44
*K*_m_ NADP^+^ (µM)	6.16	6.96	9.06	12.97
*k*_cat_ (s^−1^)	233	71	71	114
*k*_cat_/*K*_m_ G6P (s^−1^·M^−1^)	6.05 × 10^6^	2.85 × 10^6^	4.62 × 10^6^	2.02 × 10^6^
*k*_cat/_*K*_m_ NADP^+^ (s^−1^·M^−1^)	37.82 × 10^6^	10.20 × 10^6^	7.83 × 10^6^	8.78 × 10^6^

The parameters in each case were obtained from three independent experiments and from different enzyme purifications.

#### 2.2.2. Stability Characterization of Recombinant Human G6PD Enzymes

**Figure 3:**
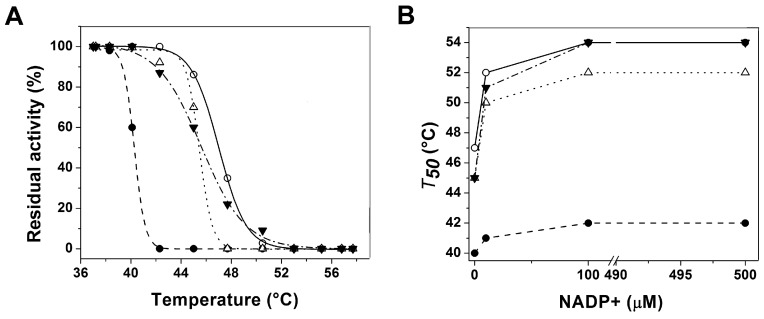
Stability characterization of recombinant human G6PD enzymes. (**A**) Thermal inactivation assays of WT-G6PD (o) and the variants G6PD Durham (●), Santa-Maria (Δ) and A+ (▼). The *T*_50_ (temperature where 50% of its original activity is retained) after 20 min incubation at different temperatures is shown; (**B**) *T*_50_ for each variant is plotted against different NADP^+^ concentrations.

Stability of the active site of WT-G6PD as well as the different G6PD variants were analysed by thermal inactivation assays. As shown in Figure 3A, the *T*_50_ values obtained without NADP^+^ were 40.3, 45.5 and 45.5 °C for the G6PD Durham, Santa-Maria and A+, respectively (Table 3). It is interesting to note that both G6PD Santa-Maria and G6PD A+ variants were more heat-resistant, with 3 °C below the value obtained for WT-G6PD. However, the Class I G6PD Durham variant was the most thermolabile protein, with a *T*_50_ of 40.3 °C, which is 8 °C lower with respect to the WT-G6PD enzyme (Table 3).

We also performed thermal inactivation assays in the presence of different concentrations of NADP^+^ (0–500 µM). The protective effect of NADP^+^ as stabilizer in thermal stability is plotted in Figure 3B, with the transition temperature *T*_50_ calculated for each variant. As shown, the thermal stability was increased about 7 °C for the WT-G6PD, Santa-Maria and A+ variants with a slight difference between variants with respect to WT-G6PD. However, for G6PD Durham variant the protective effect of NADP^+^ was not observed, because the thermal stability was increased only 2 °C compared to the WT-G6PD. In all the assays, the WT-G6PD was more stable than the variants involved in this study.

**Table 3:** Determination of thermal inactivation (*T*_50_) and melting temperature (*T*_m_) of recombinant human G6PD enzymes.

G6PD	*T*_50_ (°C)	*T*_m_ (°C)
WT	48.8 ± 0.2	54.8 ± 0.3
Durham	40.3 ± 0.3	49.9 ± 0.4
Santa-Maria	45.5 ± 0.4	54.8 ± 0.3
A+	45.6 ± 0.3	55.8 ± 0.4

Thermal inactivation (*T*_50_) assays of WT G6PD and the variants. In all cases, 200 ng of total protein was used. Thermal unfolding (*T*_m_) of WT G6PD and the variants (0.8 mg/mL) in 25 mM NaPO4 pH 7.4 was monitored by recording the change in CD signal at 222 nm at different temperatures ranging from 20 to 90 °C.

**Figure 4:**
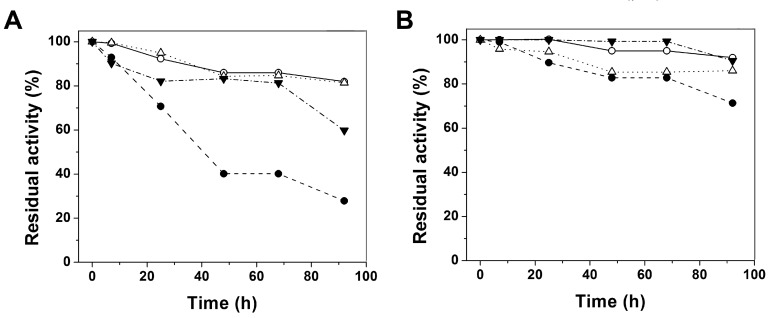
Stability of WT-G6PD and G6PD Durham, Santa-Maria and A+ variants. (**A**) Stability properties of WT-G6PD (o) and the variants G6PD Durham (●), Santa-Maria (Δ) and A+ (▼) on the time-courses without and with (**B**) NADP^+^ incubated at 37 °C. In all cases, 200 ng/mL was used to measure the activity, and the assays were performed in triplicate; standard errors were lower than 5%.

Another way to evaluate the possible changes caused by point mutations for the three clinical G6PD variants was the loss of activity during incubation times. These assays were performed at physiological temperature and in presence of physiological concentration (10 µM) or absence of NADP^+^ (Figure 4A,B). The G6PD Durham was the most thermolabile protein with loss of activity around 73% (Figure 4A). Unexpectedly the G6PD A+ was the second more thermolabile protein with a loss of 40% compared with the initial activities for WT-G6PD. When the variants were incubated with the physiological NADP^+^ concentration (10 µM), the protective effect of the NADP^+^ was observed in all of the variants (Figure 4B). However, the Class I G6PD Durham was again, the most thermolabile enzyme, with a loss of activity around 30% compared to the WT-G6PD. It is important to mention that this assay was performed at 4 and 25 °C without loss of activity.

### 2.3. Structural Characterization of Recombinant Human G6PD Enzymes

#### 2.3.1. Analysis of Secondary Structure and Thermal Stability of Recombinant Human G6PD Enzymes

**Figure 5:**
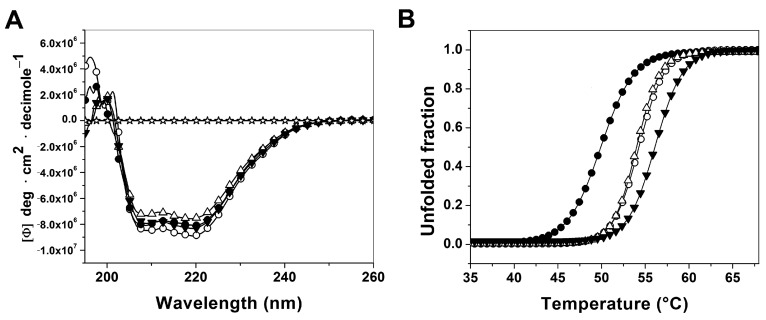
Structural characterization of recombinant human G6PDs enzymes. (**A**) Far-UV CD spectra of WT-G6PD (o) and the variants G6PD Durham (●), Santa-Maria (Δ) and A+ (▼). The experiments were performed in triplicate; standard errors were less than 4%. (**B**) Thermal stability and unfolding of human G6PD enzymes. The *T*_m_ (melting temperature midpoint of the transition values) was calculated as previously reported [7,21].

In order to determine if the diminished purification yield and the decrease in catalytic efficiency of all the variants enzymes were due to the disruption of the secondary structure of the protein, the enzymes were evaluated by circular dichroism (CD). As shown in the Figure 5A, all the variants involved in this work (Class I to III) show the same pattern and intensity of CD spectra respect to WT-G6PD. The protein stability was also studied by employing thermo stability assays. The global stability of all proteins was followed by the change in the CD signal at 222 nm when the temperature was increased. The increase in temperatures induces denaturation of all the proteins, showing a two-state process with a *T*_m_ (melting temperature midpoint of the transition) of 54 °C for the WT-G6PD enzyme and for the G6PD Santa-Maria and A+, respectively (Figure 5B; Table 3). While the G6PD Durham showed a *T*_m_ of 50 °C, showing a decrease of 5 °C respect to WT-G6PD (Table 3). This difference is consistent with that previously reported for the Class I G6PD Nashville enzyme (*T*_m_ of 50 °C), where a decrease of 5 °C was found regarding WT-G6PD.

#### 2.3.2. Analysis of Conformational Changes of Recombinant Human G6PD Enzymes

**Figure 6:**
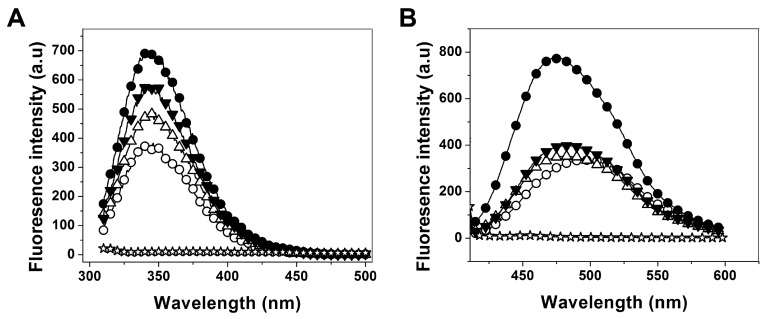
Conformational changes of recombinant human G6PD enzymes. (**A**) Fluorescence emission spectra and (**B**) ANS fluorescence emission spectra of WT-G6PD (o) and the variants G6PD Durham (●), Santa-Maria (Δ) and A+ (▼). The experimental conditions for all the experiments are described in the Materials and Methods section.

Intrinsic fluorescence and ANS assays were performed to evaluate the global structural alterations of the proteins. Fluorescence emission spectra for all the variants were increased with respect to WT-G6PD when they were analyzed at the same protein concentration (Figure 6A). The fluorescence intensity for the G6PD Durham enzyme was twice increased respect to WT-G6PD. While, the G6PD Santa-Maria and A+ the fluorescence intensity spectra were increased 1.4-fold and 1.6-fold, respectively, compared to the WT-G6PD enzyme. The variations found in intrinsic fluorescence intensity for the G6PD variant enzymes suggested slight conformational changes at fluorophore level of these proteins. The latter was confirmed by ANS assays.

To evaluate possible structural alterations in G6PD Durham, Santa-Maria and A+ variants, we analyzed the binding capacity of ANS [7,21]. The results indicate that the fluorescence emission spectrum with ANS was 2.6-fold higher for G6PD Durham variant than the WT-G6PD (Figure 6B), while the G6PD Santa-Maria and A+ variants showed a slight increase of the ANS spectra with respect to WT-G6PD. Moreover, the maximal fluorescence intensity of spectrum of ANS in WT-G6PD was recorded at 492.5 nm, whereas in the G6PD variant enzymes (except the Durham variant) the maximal fluorescence intensity with ANS was recorded at 482.5 nm (Figure 6B) (10 nm less than the WT-G6PD); notably in the Durham variant, the maximal fluorescence intensity with ANS was recorded at 476.5 nm. This represents a displacement in the maximal fluorescence intensity with ANS of 10–16 nm in the G6PD variants. In both, the increased fluorescence spectra and the shift to the blue region indicate that the ANS found more and buried hydrophobic pockets in the G6PD variant enzymes.

## 3. Discussion

G6PD deficiency is usually diagnosed through hematological studies and the severity of the symptoms. The classification is based on the level of G6PD residual enzymatic activity in red cells. Several mutations causing the disease have been identified, and the measurements of enzyme activity in red cells extracts give a reasonable indication of the altered kinetic constants of G6PD protein due to these mutations. Even so, it is not known if these alterations are due to a decrease in the catalytic efficiency, deterioration in the folding or stability of the protein [20,22]. For this reason, in order to obtain a clearer view, at the molecular, functional, and structural levels, of the basis of this enzymopathy, we characterized another three naturally G6PD variants: the Class I G6PD Durham, Class II G6PD Santa-Maria and Class III G6PD A+, comparing them with respect to the WT-G6PD enzyme.

The expression of all the proteins was performed in a heterologous expression system in genetically modified *E. coli* BL21(DE3)Δ*zwf*::kan^r^ cells. Despite the fact that all mutants were expressed under optimal conditions, they exhibited lower specific activities than the WT enzyme (Figure 2A). Furthermore, all the G6PD variants showed lower purification yields compared with those obtained for the WT-G6PD (Table 1). This phenomenon indicates that the mutations of the three pathological variants that are located at different parts of the three-dimensional structure of the protein have a negative effect on the expression of G6PD.

The alterations in the specific activity obtained for the proteins and the low purification yield for the G6PD variants were consistent with the kinetic parameters of the purified enzymes. The kinetic parameters determined for the G6PD Durham and Santa-Maria showed a decrease in catalysis. However, the mutants have a higher affinity for substrate G6P with respect to the WT-G6PD enzyme (Table 2). This decrease in *K*_m_ value was also observed in the mutant Union (Class I) [22], Andalus (Class II) [22] and Valladolid (Class II) [7]. Although this change seems favorable, at saturation levels of G6P, these variant enzymes have a diminished catalytic efficiency; we suggest that the higher affinity for G6P substrate is a compensatory mechanism due to the decrease of the catalytic efficiency. Nonetheless, it is striking that both the Class II and III G6PD Santa-Maria and A+ variants saw a decrease in catalytic efficiency of 70% and 50%, respectively, in relation to the WT-G6PD; it is also noteworthy that these variants are not associated with chronic hemolytic anemia or with acute hemolytic anemia [16].

To assess whether the diminished purification yields and the decreased catalytic efficiency was due to alterations in the secondary structure or at the global stability level of the proteins, CD studies were performed. The results (Figure 5A) indicated that all the mutants showed no significant changes at the secondary structure level. The unfolding in response to thermal denaturation of the three G6PD variants (Figure 5B) showed that they are more thermosensible with respect to WT-G6PD. These values are in concordance with the previously reported for the variants Class I, II and III G6PD Nashville, Valladolid and Mexico City, respectively [7]. Besides, the thermal inactivation of all the variants with increasing concentrations of NADP^+^ as stabilizer showed that the Class II, III and WT-G6PD enzymes were more thermo-resistant (Figure 3B). However, the protective effect was not observed in the Class I Durham, which is in concordance with what previous reports described for other Class I variants, such as G6PDs Wisconsin, Fukaya, Campinan, Plymouth, Yucatan, and Nashville [7,19,20,23]. These differences in thermal inactivation and the protective effect of NADP^+^ indicate that theses enzymes are more sensitive to temperature denaturation and suggest that the G6PD Durham, Santa-Maria and A+ variants are more unstable and relaxed in the active site.

**Figure 7:**
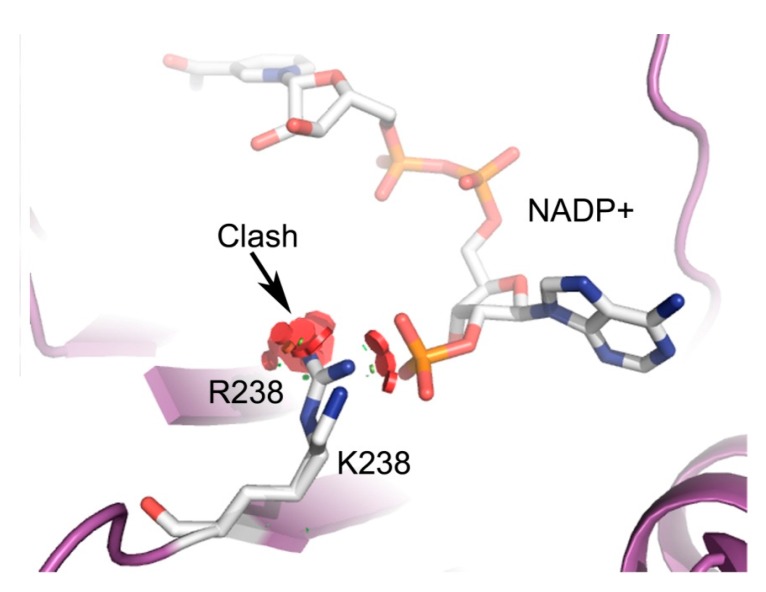
Schematic representation of the region of Class I G6PD Durham (PDB code 2BH9) [17]. We added *in silico* the Arg (R) in the Lys (L) amino acid residue at 238 positions. As indicated with the arrow, one of the rotamers of R clashes with structural NADP^+^. Modeled with PyMOL [18].

Finally, the intrinsic fluorescence and ANS assays showed that all mutants included in this study have more and buried hydrophobic regions exposed to solvent than the WT-G6PD enzyme (Figure 6B), suggesting that these enzymes have lower structural rigidity. This finding was most evident in the Durham variant. The change in the fluorescence intensity for the Class I G6PD Durham variant (Figure 6A) probably is due to the fact that the mutation K238R is located near to the structural NADP^+^ binding site (Figure 7). The replacement of Lys by Arg amino acid residue implies an increase in surface area of the side chain of 1.12-fold (200 to 225 Å^2^, respectively) [24]. Most likely, this replacement could provoke steric conflicts that cause that the arginine side chain to rotate away from structural NADP^+^, and consequently lead to a less stable NADP^+^-bound form.

## 4. Materials and Methods

### 4.1. Construction and Cloning of WT-G6PD and Durham, Santa-Maria and A+ Variants

The desired mutations in G6PD variants were generated by site-directed mutagenesis as previously reported [7]. Specific mutagenic primers were designed to create the G6PD gene mutants from humans, access number in GenBank (No. NM_001042351). Table 4 shows the primers employed to obtain desired mutations in G6PDs Durham (K238R), Santa-Maria (N126D/D181V) and A+ (N126D).

**Table 4:** Primers used in this study.

Primer Name	Primer Sequence
Durham K238R	5′-CTCACCTTCA**G**GGACCC-3´
	5´-GGGTCC**C**TGAAGGTGAG-3´
Santa-Maria N126D	5´-AGCCACATG**G**ATGCCCTCCAC-3´
	5´-GTGGAGGGC**A**TCCATGTGGCT-3´
Santa-Maria D181V	5´-AGAGCTCTG**T**CGGCTGTCCA-3´
	5´-TGGACAGCCGG**A**CAGAGCTCT-3´
A+ forward N126D	5´-AGCCACATG**G**ATGCCCTCCAC-3´
	5´-GTGGAGGGC**A**TCCATGTGGCT-3´

The letter in bold and underline indicates the mutagenic site.

The purified PCR products were ligated to pJET 1.2 vector (CloneJET PCR Cloning Kit; Thermo Scientific, (Hudson NH, USA)) and the ligation product was used to transform into competent *Escherichia coli* TOP-10 cells. Plasmidic DNA of each mutant was isolated using a plasmid miniprep kit (QIAGEN^®^, (Valencia, CA, USA)) and fully sequenced to ensure the fidelity and desired mutation of the gene. The pJET 1.2 vector containing the verified sequence for each mutant *g6pd* gene was digested with restriction enzymes *Nde*1 and *Bpu11021* and sub-cloned into the pET-3a plasmid (Novagen, Madison, WI, USA), which were named pETg-Durham, pETg-Santa-Maria, and pETg-A+.

### 4.2. Expression and Purification of Recombinant Human G6PD Enzymes

In order to find the optimal expression conditions of G6PD soluble proteins, we performed expression assays for each G6PD mutant in *E. coli* BL21(DE3)Δ*zwf*::kan^r^. For this purpose, expression conditions were followed as previously described [7]. We used three different temperatures and isopropyl-β-d-thiogalactopyranoside (IPTG) concentrations. At 2, 6, 12 and 18 h, the samples were taken; the cells were harvested, resuspended in lysis buffer, and disrupted by sonication [7]. The crude extracts were clarified by centrifugation and the supernatants were used to calculate specific G6PD activity. Subsequently, 2 L of Luria Bertani (LB) medium containing 100 μg/mL of ampicillin plus kanamycin were used to grow the G6PD variants at the optimal expression conditions The cell lysis, clarification of crude extracts, and the purification procedure for all the variants were performed as previously described [7], except for Class I G6PD Durham variant, which was purified using only the 2′,5′-ADP Sepharose-4B affinity column. The purity of the recombinant enzymes was confirmed on 12% SDS-PAGE gels revealed with Coomassie brilliant blue (R-250).

### 4.3. Functional Characterization of Recombinant Human G6PD Enzymes

#### 4.3.1. Steady-State Kinetic Experiments

The enzymatic activity of WT-G6PD and its variants was determined spectrophotometrically by monitoring the reduction of NADP^+^ at 340 nm [6,7,25]. The reaction was initiated with the addition of 200 ng/mL of each variant enzyme. Initial velocity data were obtained by varying one substrate (2.5 to 200 µM), while the second substrate was fixed at saturating concentration. The *k*_cat_ for each variant was calculated according to data previously reported [7,25]. One international unit (IU) of G6PD activity is the amount of enzyme required to produce 1 µmol of NADPH per minute under the assay conditions.

#### 4.3.2. Thermal Inactivation Assays

The human WT-G6PD enzyme and the mutants were subjected to thermal inactivation tests employed 0.2 mg/mL of each enzyme and incubated for 20 min at temperatures ranging from 37 to 61 °C as previously reported [7,25]. Then, the samples were ice-cooled and the residual enzyme activity was measured with the standard reaction mixture at 25 °C. In addition, these assays were performed at increasing NADP^+^ concentrations (0, 10, 100 and 500 µM, respectively). Enzyme stability during storage was assessed with two different concentrations of NADP^+^ (0 and 10 µM) and incubated over time at three different temperatures (4, 25 and 37 °C, respectively). For all the assays, residual enzyme activity was expressed as the percentage of the enzyme activity, taken as 100% the enzyme without incubation; the trials were repeated at least three times.

### 4.4. Structural Characterization of Recombinant Human G6PD Enzymes

#### 4.4.1. Analysis of Secondary Structure and Thermal Stability

Analysis of secondary structure, thermal stability and thermal unfolding (*T*_m_) of the WT-G6PD and the variants were evaluated by CD in a spectropolarimeter (Jasco J-810^®^, Easton, MD, USA) equipped with a Peltier thermostatted cell holder [7,21,25]. The spectral scans were done from 200 to 260 nm at 1 nm intervals. The protein thermal unfolding was at 0.8 mg/mL in phosphate buffer measured as the change in the CD signal at 222 nm in scans from 20 to 90 °C at an increasing rate of 1 °C/2.5 min. For both assays, the spectra of blanks were subtracted from those that contained the proteins.

#### 4.4.2. Analysis of Conformational Changes of Recombinant Human G6PD Enzymes

Intrinsic fluorescence and 8-anilino-1-naphthalenesulfonic acid (ANS) assays of the recombinant human WT-G6PD and G6PD variants were conducted as described formerly [7,21]. Both assays were monitored in a Perkin-Elmer LS-55 spectrofluorometer (Wellesley, MA, USA). Emission fluorescence spectra from 310 to 500 nm were recorded after excitation at 295 nm, whereas the ANS assays were conducted using an excitation wavelength of 395 nm and recording the emission fluorescence spectra from 400–600 nm. All the intrinsic fluorescence and ANS assays were conducted as described previously [7,21,25]. Background fluorescence from the buffer (blank), and buffer plus ANS was subtracted from those that contained the respective protein.

## 5. Conclusions

In conclusion, we have reported on the structural and functional characterization of three naturally occurring variants corresponding to different classes of disease severity. Although the mutations are located in different parts of the protein, they induce a decrease in catalytic efficiency, greater sensitivity to temperature denaturation, and exposure of hydrophobic pockets in the variants, probably as the result of a less compact structure in the three-dimensional structure of the protein. The degree of protein affectation by each mutation correlates with the severity of clinical manifestations reported in different patients.
